# Physiological and Proteomic Analyses Reveal the Adaptive Mechanisms of Maize Genotypes Under Potassium Deficiency

**DOI:** 10.3390/ijms27188286

**Published:** 2026-09-17

**Authors:** Xudong Yang, Haoran Qin, Fei Zou, Hui Ma, Hui Li, Yanye Ruan, Shuisen Chen

**Affiliations:** 1 College of Bioscience and Biotechnology, Shenyang Agricultural University, Shenyang 110866, Chinamahui@syau.edu.cn (H.M.); 2017500072@syau.edu.cn (H.L.); yanyeruan@syau.edu.cn (Y.R.); 2 Maize Research Institute, Hulunbuir Academy of Agricultural and Animal Husbandry Sciences, Zhalantun 162650, China

**Keywords:** maize, potassium, roots, proteomics, iTRAQ

## Abstract

Potassium (K) deficiency severely constrains maize productivity, yet the molecular mechanisms underlying genotypic differences in K tolerance remain poorly understood. A comparative physiological and iTRAQ-based quantitative proteomic analysis was conducted on two maize inbred lines with contrasting K-deficiency tolerance (Ktm, tolerant; Ksm, sensitive) following 3 days of K starvation (0 mM K), using roots as the primary analytical target. Physiological assessments revealed that under K deficiency, Ktm exhibited higher root vitality (Ktm decreased by 16.65% vs. Ksm by 31.54%) and larger root volume but lower electrolyte leakage compared with Ksm. Proteomic profiling identified 93 and 126 differentially abundant proteins (DAPs) in Ktm and Ksm, respectively. Integrative analysis indicated that Ktm responsed to K deprivation though coordinated downregulation of glycolytic enzymes, differential ROS accumulation with enhanced catalase (CAT) and peroxidase (POD) upregulation, and increased the abundances of cell wall-reinforcing proteins (dirigent proteins and cinnamyl alcohol dehydrogenase). In contrast, Ksm exhibited a less efficient stress response characterized by impaired ROS signaling, H_2_O_2_ accumulation, and compromised membrane permeability. These results suggested that under short-term K starvation, K tolerance in maize was associated more closely with the efficiency of metabolic reprogramming and ROS homeostasis than with differences in root K content. Our findings provide a mechanistic framework for understanding genotypic variation in K adaptation and identify candidate protein markers for breeding K-efficient maize varieties.

## 1. Introduction

Potassium (K), as an essential macronutrient, drives plant development by controlling water balance, activating enzymes, modulating stomatal behavior, and bolstering resilience against environmental stress [1,2]. Nevertheless, the majority of K in soil exists in fixed forms that are unavailable to plants, with merely 0.1–0.2% available for directly absorption by roots [3]. Insufficient K in the soil consequently reduces nutrient use efficiency and leads to unstable yields [4]. Moreover, K deficiency severely restricts root development and overall plant growth, representing a widespread and major obstacle to crop production [5]. Approximately 20% of the world’s arable land [6] and an estimated 40% of arable soils in China are reported to be deficient in available K [7]. Unfortunately, only about 50% of applied K fertilizer is utilized by crops, which is attributed to low K use efficiency [8]. Fortunately, sophisticated adaptive response mechanisms to K deficiency have evolved in plants. Genotypic differences in potassium uptake and use efficiency have been documented in all major crop species [3,9]. Therefore, the mechanisms underlying crop tolerance to K deficiency must be urgently revealed, and crop varieties with high K uptake and utilization efficiency need to be developed.

Among staple crops, maize (*Zea mays*) has a relatively high demand for K and is particularly prone to low K stress. It was estimated that the total K requirement per 1000 kg of grain is 13.8 kg for spring maize and reaches 15.8 kg for summer maize. Across the main maize cultivation areas in China, omitting K fertilizer led to an average yield loss of 11% to 18% [10]. In China, substantial K fertilizer inputs are required for maize production, while relatively low K uptake and utilization efficiency are exhibited by maize cultivars [11]. It is important to distinguish among K uptake efficiency (the ability to acquire K from the soil), K utilization efficiency (the ability to produce biomass per unit of K absorbed), and K-deficiency tolerance (the ability to maintain physiological function under suboptimal K supply). Maize genotypes with contrasting low K tolerance exhibited divergent responses to K deficiency. K-tolerant maize genotypes tend to maintain larger root systems and higher K^+^ uptake affinity [12]. Additionally, osmotic adjustment via soluble sugars and soluble proteins is commonly employed as a short-term adaptive response to K scarcity [12]. K transporters of the HAK/KUP/KT family have been identified as key players in K^+^ acquisition and translocation in maize [13]. Hydroponic and field trials indicated that K deficiency reduced total root length, primary root length, and maximum root number of maize seedlings by 50% to 80% [14]. In addition, transcriptomic and metabolomic analyses have revealed that phenylpropanoid biosynthesis and hormone signaling pathways were intensively rewired under low-K conditions [15].

Although there have been certain advancements in maize tolerance to low K stress, the systemic correlation between global proteomic alterations and key physiological indicators has rarely been explored in maize root subjected to K deficiency. Most previous studies have examined physiological endpoints and molecular profiles in isolation, leaving it unclear how specific changes in protein abundance systematically translate into distinct physiological outcomes. Consequently, the integrative mechanism by which proteomic reconfiguration drives genotype-specific physiological adaptation has not been systematically established in maize roots under K deficiency. To bridge these gaps, a comprehensive investigation combining physiological measurements with comparative proteomic analysis using two maize inbred lines with contrasting K-deficiency tolerance were conducted in the present study. The specific objectives were to identify genotype-discriminating proteomic alterations and integrate them with physiological responses into a model of K deficiency adaptation. We hypothesized that K-tolerant maize (Ktm) conferred superior adaptation through coordinated regulation of proteins involved in energy metabolism, osmotic adjustment, redox regulation, and cell wall remodeling. Specifically, we expected Ktm to show enhanced cell wall reinforcement and secondary metabolism (e.g., lignin biosynthesis) as a structural defense, whereas Ksm would exhibit impaired redox regulation and weakened cell wall integrity, leading to compromised root vitality. The present study not only provides novel insights into the molecular basis of maize tolerance to K deficiency at the protein level but also offers potential protein candidates that warrant further evaluation as biomarkers for marker-assisted breeding of K-efficient maize cultivars, ultimately contributing to sustainable agriculture by reducing reliance on K fertilizer inputs.

## 2. Results

### 2.1. Effect of K Starvation on Plant Growth and Root Characteristics

**Figure 1:**
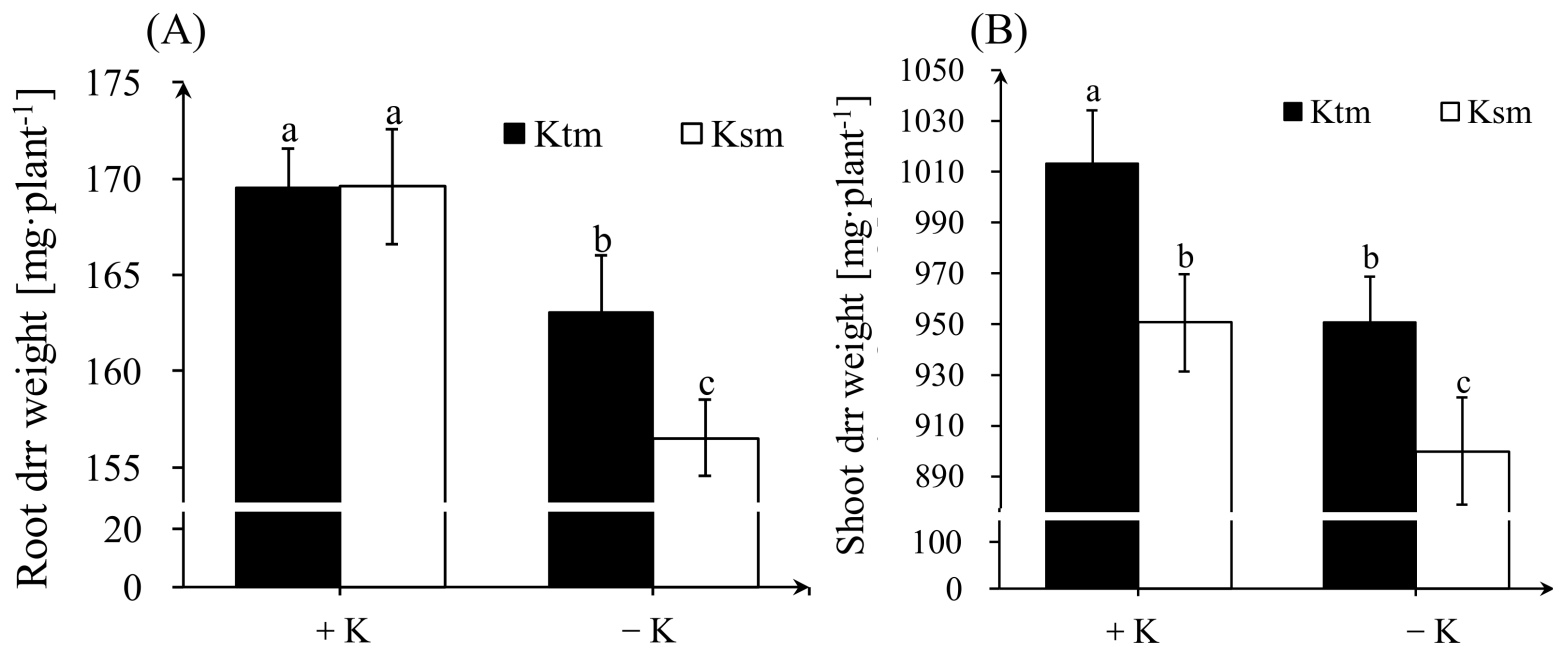
The effect of K starvation on shoot (**A**) and root (**B**) dry weight of maize genotypes with different K efficiencies. Here, +K and −K represent seedlings under high K (1.25 mM K) and K starvation (0 mM K) for 3 d, respectively. Values represent the mean ± SD of three replicates. Bars with different letters show significant differences at *p* < 0.05.

K deficiency significantly inhibited the growth of maize. The DW values of roots and shoots in maize genotypes were significantly decreased after K starvation for 3 d (Figure 1). The decrease in root DW (dry weight) of K-tolerant maize genotype (Ktm) was lower than that of K-sensitive maize genotype (Ksm) under K starvation (Figure 1A). Ktm has higher shoot DW than Ksm, no matter under K starvation or K-sufficient conditions (Figure 1B).

**Figure 2:**
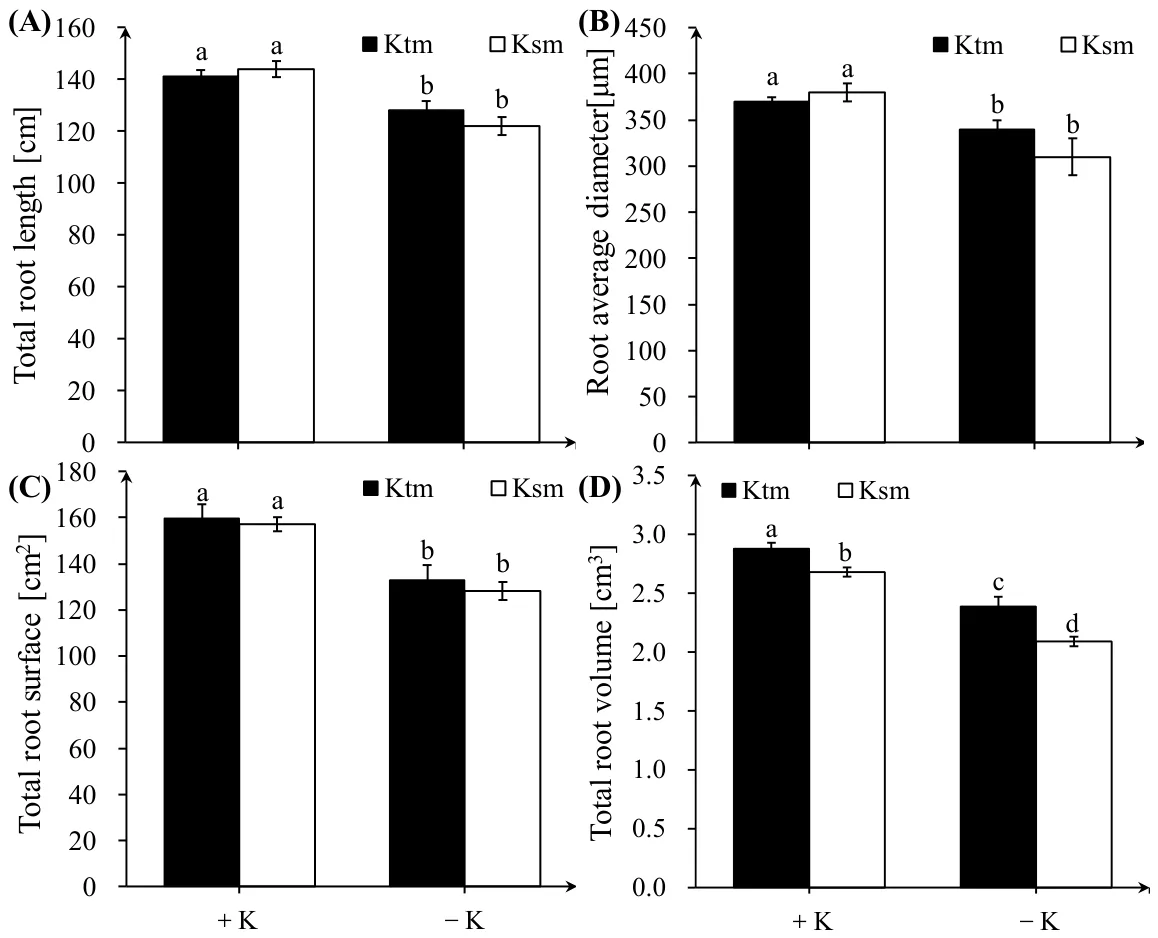
The effect of K starvation on the total root length (**A**), root average diameter (B), total root surface (**C**), and total root volume (**D**) of maize genotypes with different K efficiencies. Here, +K and −K represent seedlings under high K (1.25 mM K) and K starvation (0 mM K) for 3 d, respectively. Values represent the mean ± SD of three replicates. Bars with different letters show significant differences at *p* < 0.05.

**Figure 3:**
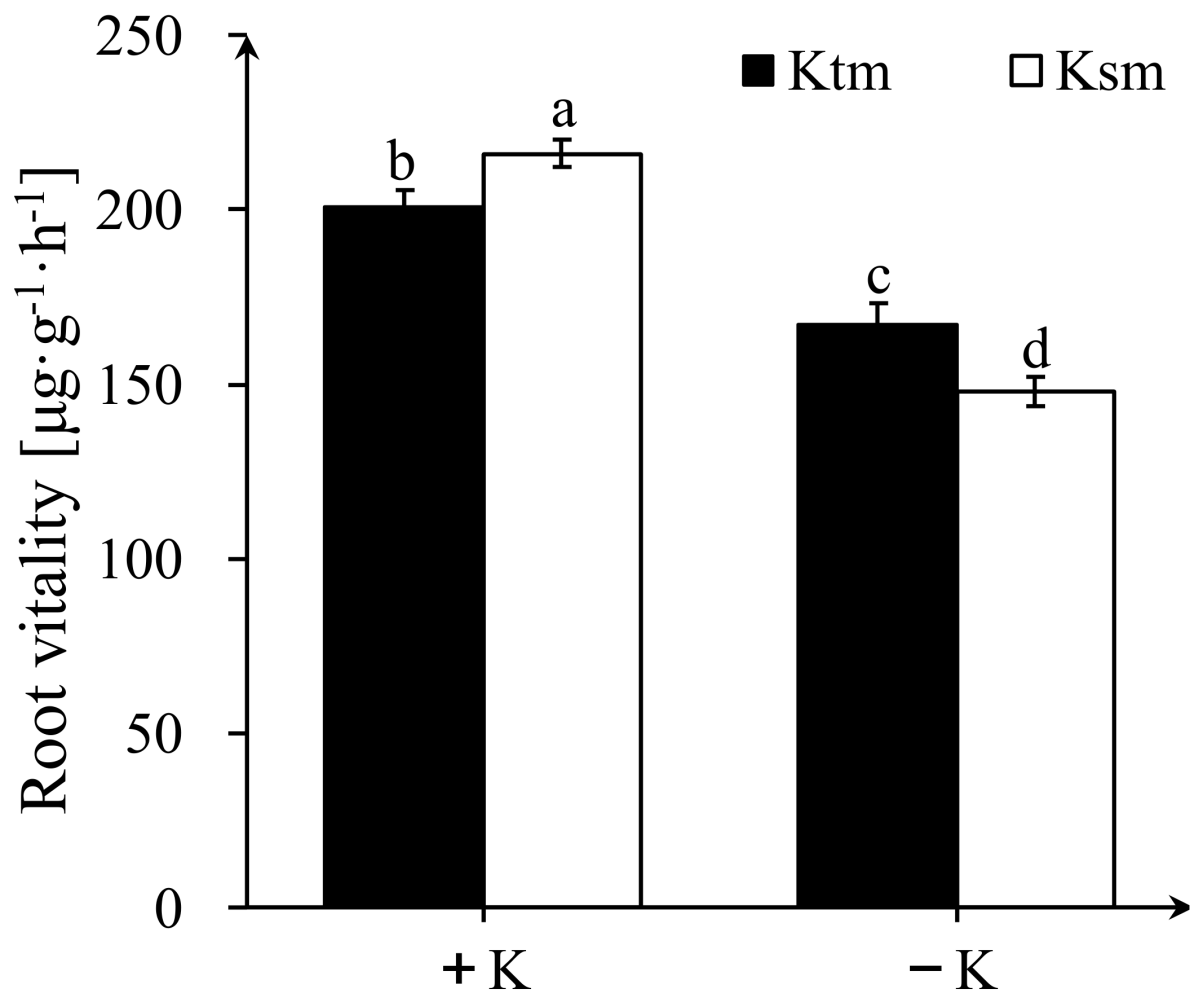
The effect of K starvation on root vitality of maize genotypes with different K efficiencies. Here, +K and −K represent seedlings under high K (1.25 mM K) and K starvation (0 mM K) for 3 d, respectively. Values represent the mean ± SD of three replicates. Bars with different letters show significant differences at *p* < 0.05.

Under K starvation for 3 d, root trait parameters including total root length, root average diameter, total root surface, and total root volume were significantly decreased both in Ktm and Ksm. However, there were no significant differences between the two maize genotypes except the total root volume (Figure 2). The K-tolerant maize genotype (Ktm) maintained a larger total root volume in response to K deficiency compared to the K-sensitive maize genotype (Ksm) (Figure 2D). K deficiency also reduced root vitality in both maize genotypes. Under K-sufficient conditions, the root vitality of Ktm was lower than that of Ksm, whereas under K starvation, Ktm exhibited higher root vitality than Ksm. K deficiency caused a decreased in root vitality of 16.65% and 31.54% in Ktm and Ksm, respectively (Figure 3).

### 2.2. Effect of K Starvation on Nutrient Uptake in the Root System

**Figure 4:**
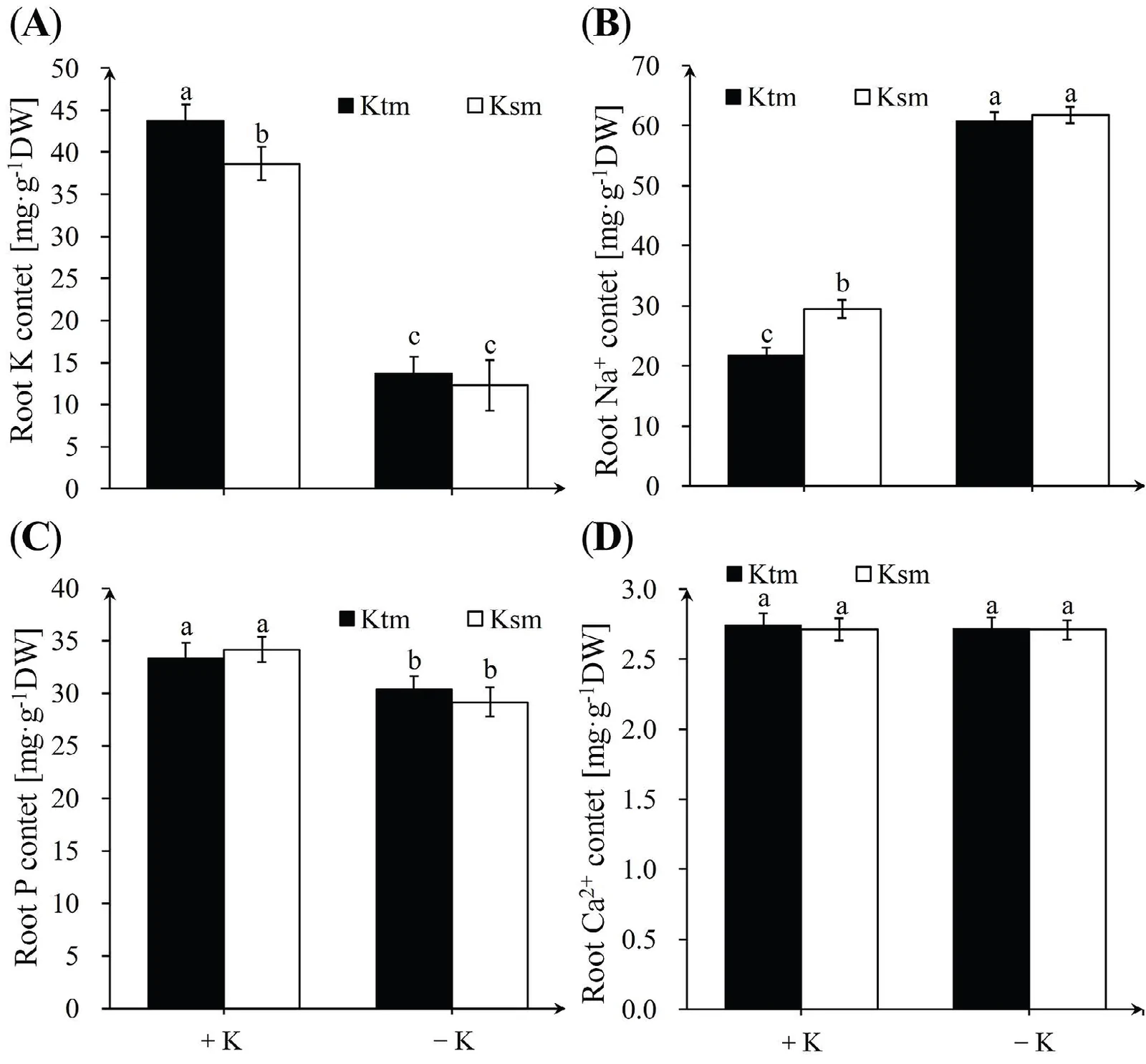
The effect of K starvation on K content (**A**), Na content (**B**), P content (**C**), and Ca content (**D**) in roots of maize genotypes with different K efficiencies. Here, +K and −K represent seedlings under high K (1.25 mM K) and K starvation (0 mM K) for 3 d, respectively. Values represent the mean ± SD of three replicates. Bars with different letters show significant differences at *p* < 0.05.

The effects of K deficiency on root K, Na, P, and Ca contents were further examined. Following 3-day K starvation, root K and P contents decreased significantly, whereas root Na content increased significantly. Root Ca content showed no difference in both maize genotypes. Nevertheless, no significant genotypic differences were observed for any of these elements under K starvation (Figure 4).

### 2.3. Effect of K Starvation on Osmotic Regulatory Substances in the Root System

**Figure 5:**
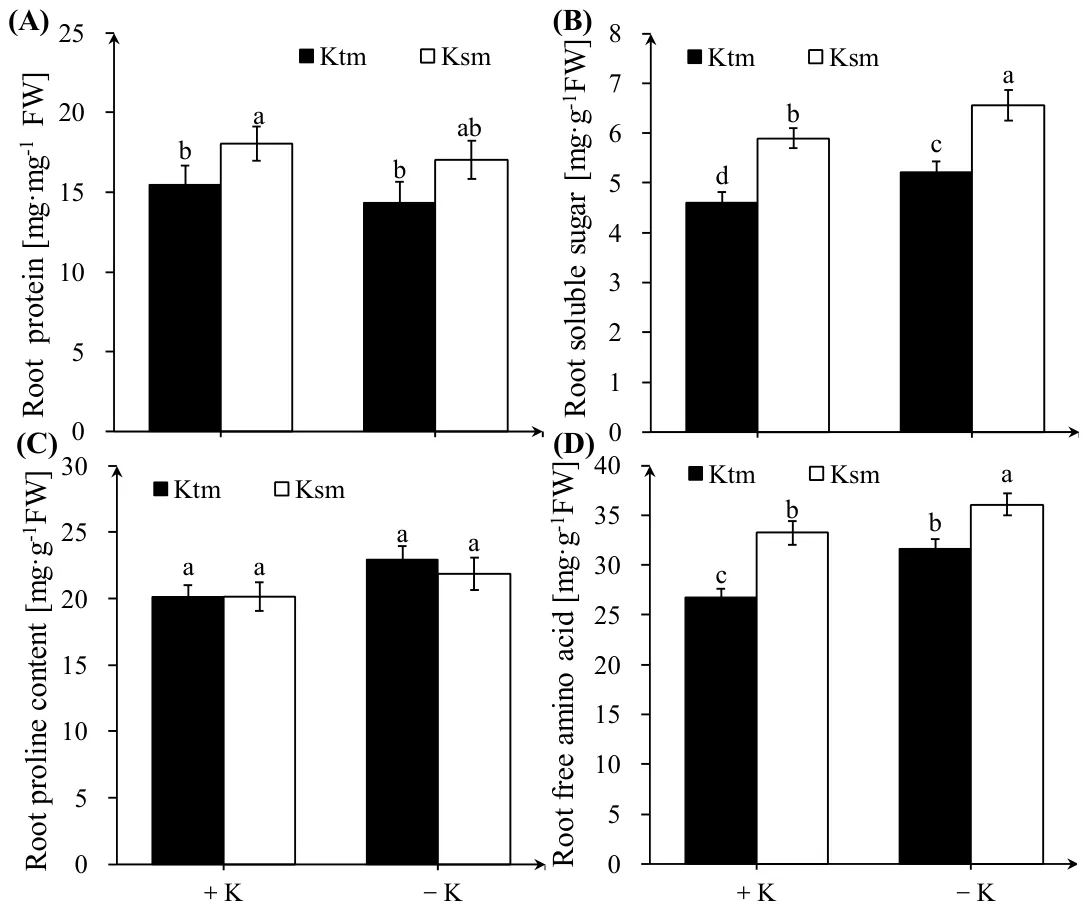
The effect of K starvation on protein content (**A**), soluble sugar (**B**), proline content (**C**), and free amino acid (**D**) in roots of maize genotypes with different K efficiencies. Here, +K and −K represent seedlings under high K (1.25 mM K) and K starvation (0 mM K) for 3 d, respectively. Values represent the mean ± SD of three replicates. Bars with different letters show significant differences at *p* < 0.05.

The changes in osmotic regulatory substances in roots following K starvation were further investigated. A 3-day K starvation period had no significant effect on root protein or proline contents in both maize genotypes (Figure 5A,C), although root protein content was lower in the K-tolerant maize genotype (Ktm) than in the K-sensitive maize genotype (Ksm) (Figure 5A). However, soluble sugar and free amino acid contents were significantly increased by K starvation in both genotypes, and both soluble sugar and free amino acid contents were present at significantly higher levels in Ksm compared with Ktm (Figure 5B,D).

### 2.4. Antioxidant Analysis of Roots Under K Starvation

**Figure 6:**
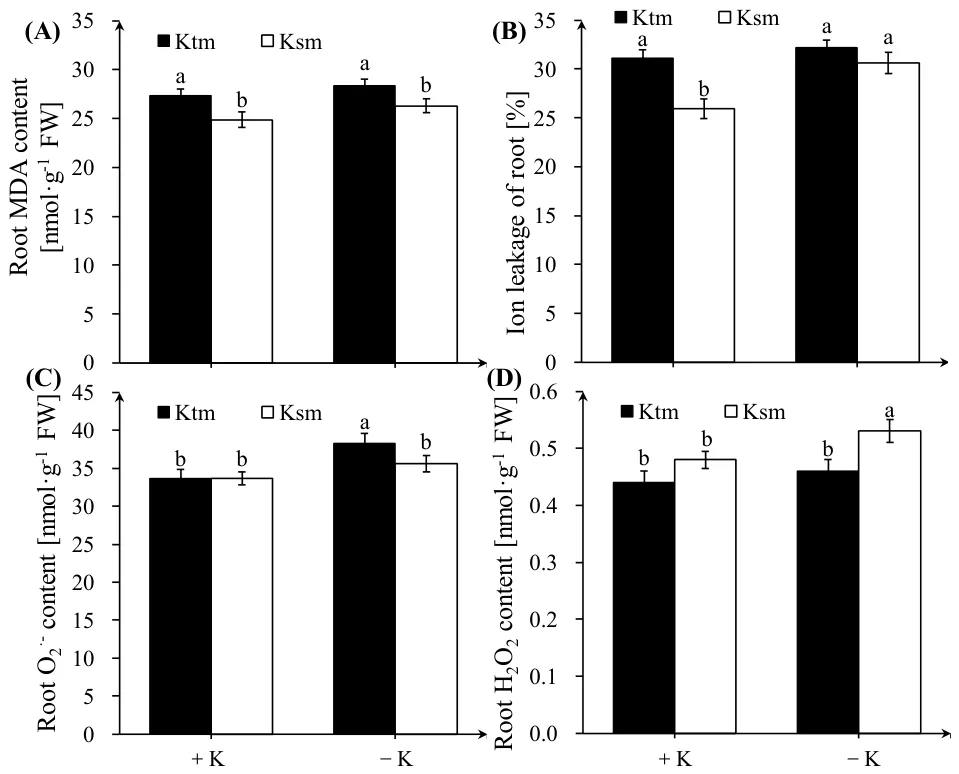
The effect of K starvation on MDA content (**A**), ion leakage (**B**), O_2_^−^ content (**C**), and H_2_O_2_ content (**D**) in roots of maize genotypes with different K efficiencies. Here, +K and −K represent seedlings under high K (1.25 mM K) and K starvation (0 mM K) for 3 d, respectively. Values represent the mean ± SD of three replicates. Bars with different letters show significant differences at *p* < 0.05.

MDA content is widely regarded as a marker of lipid peroxidation. There were no significant changes of MDA content in both maize genotypes under K deficiency, although the MDA level was higher in Ktm than in Ksm (Figure 6A). Moreover, root ion leakage was significantly increased in t Ksm after 3 days of K deprivation, while no significant alteration was found in Ktm (Figure 6B). These results suggested that K starvation-induced membrane dysfunction in maize is primarily associated with impaired membrane permeability rather than severe lipid peroxidation.

**Figure 7:**
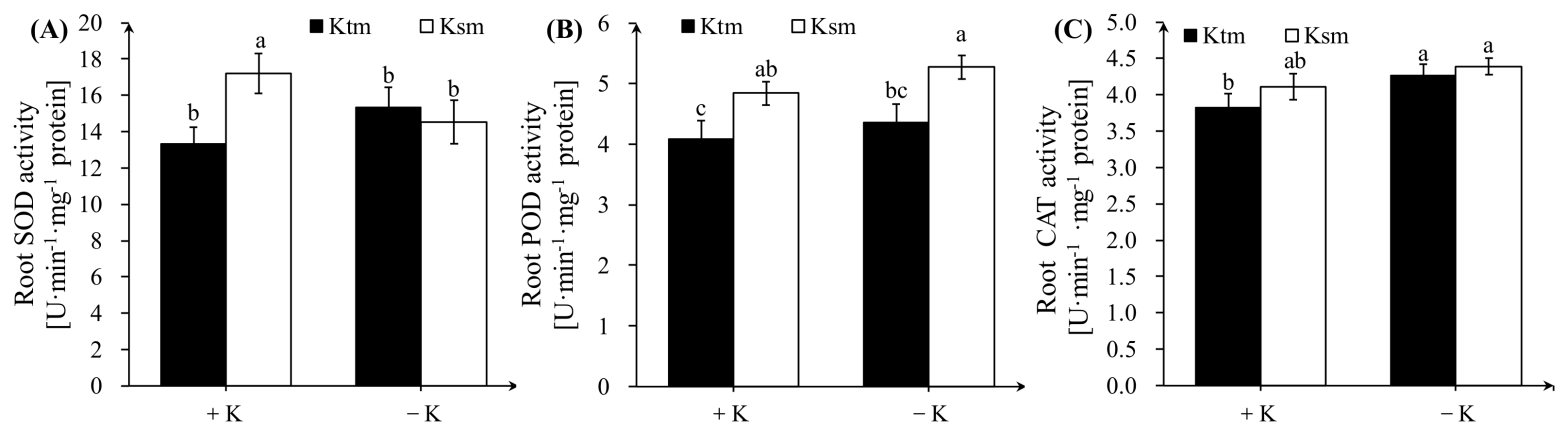
The effect of K starvation on SOD activity (**A**), POD activity (**B**) and CAT activity (**C**) in roots of maize genotypes with different K efficiencies. Here, +K and −K represent the seedling under high K (1.25 mM K) and K starvation (0 mM K) for 3 d, respectively. Values represent the mean ± SD of three replicates. Bars with different letters show significant differences at *p* < 0.05.

The accumulation of reactive oxygen species (ROS) in roots was compared between the two maize genotypes. Under K deficiency, the O_2_^−^ content was significantly increased in K-tolerant maize genotype (Ktm) (Figure 6C), and the H_2_O_2_ content was significantly increased in K-sensitive maize genotype (Ksm) (Figure 6D). Furthermore, the changes in activities of antioxidant enzymes in roots were investigated. The activity of SOD was significantly decreased in Ksm after K starvation for 3 d. In contrast, the activity of SOD was increased slightly in Ktm, but there was no significant change (Figure 7A). After K deprivation, POD activity increased slightly in both genotypes, but the change was not statistically significant. Notably, the K-sensitive maize genotype (Ksm) consistently maintained significantly higher POD activity than the K-tolerant maize genotype (Ktm), irrespective of K deficiency treatment (Figure 7B). After 3 days of potassium starvation, CAT activity increased in both maize genotypes, but the increase was significant only in Ktm. However, no significant change was observed in Ksm (Figure 7C).

### 2.5. Proteomic Analysis of Roots Under K Starvation

**Figure 8:**
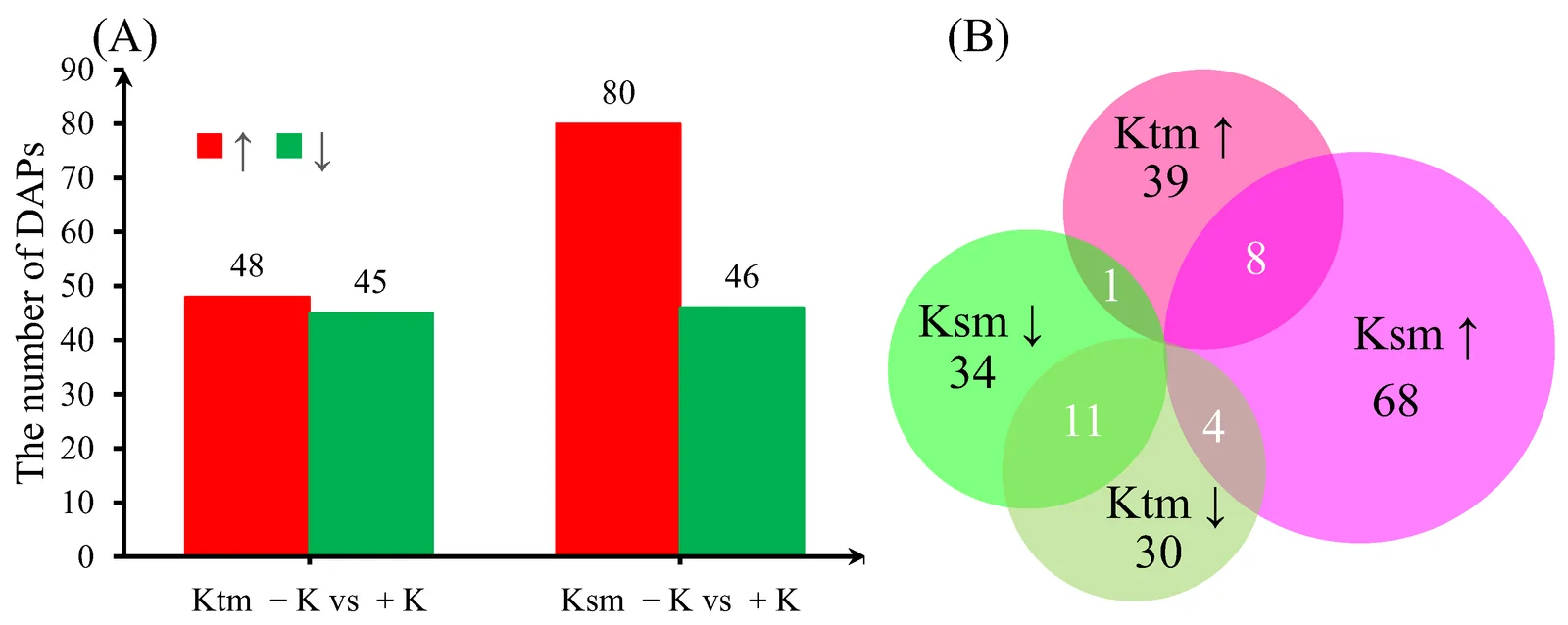
The number of DAPs in response to K starvation in Ktm and Ksm, respectively (**A**); the commonly identified DAPs (overlap area) of Ktm and Ksm (**B**).

Plant roots constitute the primary site for K^+^ uptake and perception. To gain insight into the molecular responses to K deficiency, a quantitative proteomic analysis using iTRAQ labeling was conducted on root tissues of both maize genotypes following a 3-day potassium starvation. Proteomic profiling identified 93 DAPs in Ktm (45 decreased in protein abundance and 48 increased in protein abundance) (Figure 8A and Supplementary Table S1), whereas 126 DAPs were detected in Ksm (46 decreased in protein abundance and 80 increased in protein abundance) (Figure 8A and Supplementary Table S2), indicating a more extensive proteomic reprogramming in the K-sensitive maize genotype (Ksm) under K deficiency. There were 24 DAPs identified both in Ktm and Ksm, in which 19 DAPs with the same pattern of alteration including 8 DAPs increased in protein abundance and 11 DAPs decreased in protein abundance. One DAP, pirin-like protein, was increased in Ktm, whereas four DAPs decreased in Ktm. All five DAPs exhibited opposite abundance patterns in Ksm under K deficiency (Figure 8B).

### 2.6. GO and KEGG Enrichment Analysis of DAPs

In the K-tolerant maize genotype (Ktm), 45 DAPs with decreased in protein abundance were significantly assigned to 40 GO terms. Of these, 38 GO terms were biological process categories, and 2 GO terms were cellular component categories. In total, 48 DAPs with increased in protein abundance were significantly assigned to 12 GO terms. Of these, 8 GO terms were biological process categories, and 4 GO terms were molecular function categories (Supplementary Table S3). In the K-sensitive maize genotype (Ksm), 46 DAPs with decreased in protein abundance were significantly assigned to 45 GO terms. Of these, 39 GO terms were biological process categories, and 6 GO terms were molecular function categories. In total, 80 DAPs with increased in protein abundance were significantly assigned to 55 GO terms. Of these, 31 GO terms were biological process categories, 12 GO terms were cellular component categories, and 12 GO terms were molecular function categories (Supplementary Table S3).

The 45 decreased protein abundance and 48 increased protein abundance DAPs of Ktm were significantly enriched in 12 pathways and 7 pathways, respectively (Table 1). The 46 decreased and 80 increased protein abundance DAPs of Ksm were significantly enriched in 9 pathways and 19 pathways, respectively (Table 1).

### 2.7. Protein–Protein Interaction Network of Common Identified DAPs in Both Genotypes

**Figure 9:**
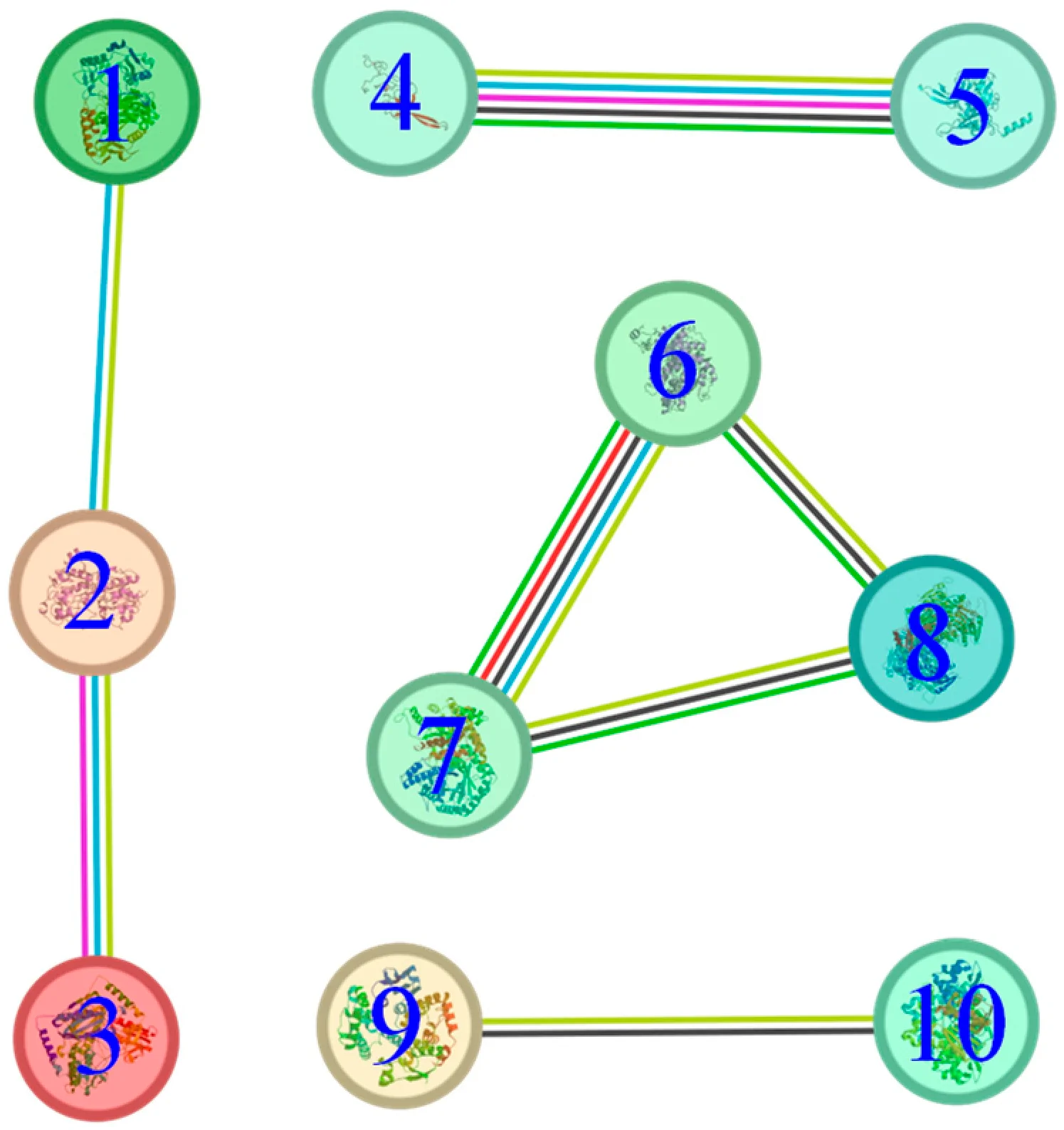
Protein interaction network of commonly identified DAPs both in Ktm and Ksm in response to K deficiency. The network was built using STRING software (https://string-db.org/) with the *Zea mays* database, applying a medium confidence interaction score threshold of 0.4. The nodes represent the DAPs, and the line color indicates the type of interaction evidence. The DAP names are as follows: 1. Phosphoenolpyruvate carboxykinase (Ktm ↑ and Ksm ↑); 2. Formate dehydrogenase (Ktm ↑ and Ksm ↑); 3. Aminodeoxychorismate lyase (Ktm ↑ and Ksm ↑); 4. 40S ribosomal protein S11 (Ktm ↓ and Ksm ↑); 5. 40S ribosomal protein S4 (Ktm ↓ and Ksm ↑); 6. Adenosylhomocysteinase (Ktm ↓ and Ksm ↑); 7. 5-Methyltetrahydropteroyltriglutamate-homocysteine S-methyltransferase (Ktm ↓ and Ksm ↑); 8. D-3-phosphoglycerate dehydrogenase (Ktm ↑ and Ksm ↑); 9. Glutathione transferase (Ktm ↓ and Ksm ↓); 10. Herbicide safener binding protein (Ktm ↑ and Ksm ↑). Here, ↑ and ↓ represent increased and decreased protein abundance of DAPs, respectively.

In the protein–protein interaction of common identified DAPs both in Ktm and Ksm in response to K deficiency, 2 networks and 2 pairs of interacting proteins were formed (Figure 9). Network 1 consisted of three DAPs (phosphoenolpyruvate carboxykinase, formate dehydrogenase, and aminodeoxychorismate lyase), all of which were increased in abundance in both genotypes. In another interaction network, 2 DAPs (40S S4 and S11) were decreased in Ktm but increased in Ksm, while the remaining DAP (adenosylhomocysteinase) was increased in both maize genotypes (Figure 9). Both DAPs (5-methyltetrahydropteroyltriglutamate-homocysteine S-methyltransferase and adenosylhomocysteinase) showed opposite regulation between genotypes in one of the protein–protein interaction pair. In another protein–protein interaction pair, glutathione transferase and herbicide safener binding protein, showing similar alterations in the two maize genotypes (Figure 9).

Notably, the translational machinery (ribosomal proteins) and methyl cycle (adenosylhomocysteinase and homocysteine methyltransferase) modules were both downregulated in Ktm but upregulated in Ksm. This mirror image regulation at the network level strongly suggested that Ktm employed a holistic strategy of repressing energetically expensive processes to conserve resources, whereas Ksm failed to coordinate these modules, resulting in futile energy expenditure. In contrast, the anaplerotic (phosphoenolpyruvate carboxykinase and formate dehydrogenase) and detoxification (GST) modules showed common or distinct genotype modulations that underpinned the conserved redox maintenance and genotype-specific membrane integrity, respectively. Thus, the PPI network transformed a list of individual DAPs into a systemic view of stress response strategies, highlighting that the coordination among pathways, rather than the change of any single protein, dictated K-deficiency tolerance.

## 3. Discussion

Plant proteomics bridges basic research and agricultural practice by uncovering the molecular underpinnings of abiotic stress adaptation and offering a holistic strategy for breeding stress-tolerant crops. In the present study, a comparative physiological and proteomics-based study was conducted to elucidate how maize copes with K deficiency.

### 3.1. DAPs (Differentially Abundant Proteins) Involved in Energy Metabolism

The differential proteomic responses highlight a fundamental dichotomy in metabolic strategy between the two genotypes. Under K starvation, the K-tolerant maize genotype (Ktm) showed coordinated downregulation of multiple glycolytic enzymes, including triose phosphate isomerase, glyceraldehyde-3-phosphate dehydrogenase, fructose-bisphosphate aldolase, enolase 1, pyruvate kinase, and fermentative enzymes (alcohol dehydrogenase 1 and pyruvate decarboxylase) (Table 2). K deficiency also directly inhibited the activity of pyruvate kinase in *Arabidopsis* [16]. Metabolic downregulation has been observed as a conserved energy conservation strategy in plants facing nutrient deprivation [17,18]. Consider that the differential DW accumulation of shoot and root to K starvation, Ktm might conserve energy by reducing glycolytic flux, potentially channeling limited resources toward stress resilience rather than immediate growth. This interpretation was consistent with the “growth defense trade-off” theory, wherein tolerant genotypes allocate resources to stress resilience at the expense of immediate growth [19,20]. Although certain glycolytic enzymes (e.g., glucose-6-phosphate isomerase) were downregulated in Ksm, several energy-generating pathways were induced, including starch degradation (alpha 1,4 glucan phosphorylase), oxidative phosphorylation (ATP synthase subunit gamma), and fatty acid β oxidation (long-chain CoA ligase, acetyl CoA C acyltransferase) (Table 2). These results suggested that Ksm attempted to compensate for reduced glycolysis by tapping into alternative carbon sources. Ktm maintained significantly higher root vitality than Ksm after 3 days of K deprivation, with a decrease of only 16.65% relative to 31.54% in Ksm (Figure 3). These contrasting patterns led us to hypothesize that Ktm favored a suppression-based energy conservation, whereas Ksm engaged a reserve-mobilizing and ATP-sustaining strategy under K deficiency. However, we noted that this interpretation was derived from changes in protein abundance rather than direct flux measurements, and alternative explanations cannot be excluded. In oil palm leaves, the K deficiency-mediated suppression of glycolytic flux was mitigated by enhanced flux through the tyrosine catabolic pathway [21]. The maintenance of root vitality, as measured by TTC reduction, was consistent with sustained respiratory capacity in Ktm. Previous studies have suggested that maintenance of root respiratory activity was a key determinant of nutrient-stress tolerance, as it sustained energy-dependent uptake and assimilatory processes [22]. However, we acknowledge that TTC reduction is an indirect measure of respiratory function, and direct measurements of oxygen consumption or ATP levels would provide more definitive evidence.

### 3.2. DAPs (Differentially Abundant Proteins) Involved in Osmotic Adjustment

Both genotypes accumulated soluble sugars and free amino acids in response to K deficiency, yet Ksm consistently accumulated significantly higher levels of both osmolytes than Ktm (Figure 5B,D). In Ktm, the increased abundance of acetylornithine transaminase and acetylornithine deacetylase (Table 3) suggested a potential redirection of nitrogen flux toward the ornithine pathway, which could favor the biosynthesis of proline and polyamines. However, the latter possibility required direct polyamine measurements for confirmation. Proline is known to function not only as a compatible osmolyte but also as a ROS scavenger and protein stabilizer [23,24,25]. However, since proline content did not increase significantly under K starvation (Figure 5C) and polyamines were not directly measured in this study, this interpretation remained speculative and required further experimental validation. In Ksm, increased protein abundance of glutamate decarboxylase directs glutamate toward γ-aminobutyric acid (GABA) production [26], while simultaneously decreasing glutamine synthetase, which may impair overall nitrogen homeostasis (Table 3). The increased abundance of ferredoxin-dependent glutamate synthase and glutamate dehydrogenase suggested a highly perturbed nitrogen reassimilation cycle (Table 3), likely driven by the excessive carbon skeletons derived from the upregulated alpha 1,4 glucan phosphorylase and transketolase (Table 3). Ksm uniquely upregulated beta fructofuranosidase 1, and alpha 1,4 glucan phosphorylase increased soluble sugar accumulation. Under stress, soluble sugars accumulate as a consequence of both increased sucrose hydrolysis and decreased utilization in growth. However, excessive soluble sugar accumulation can reflect metabolic stasis rather than adaptive osmoregulation [27]. The higher accumulation of soluble sugars in Ksm (Figure 5B), coupled with upregulation of starch-degrading enzymes, might reflect an imbalance between carbon production and utilization under stress, although whether this represented adaptive osmoregulation or metabolic dysregulation requires further investigation. These results suggested that Ktm prioritized nitrogen efficient osmotic adjustment, whereas Ksm engages a broader but potentially more carbon costly strategy.

### 3.3. DAPs (Differentially Abundant Proteins) Involved in Redox Regulation

Unexpectedly, malondialdehyde (MDA) content, a classical marker of lipid peroxidation, did not change significantly in either genotype under K starvation (Figure 6A). However, root ion leakage (electrolyte conductance) increased dramatically and exclusively in Ksm (Figure 6B). These results indicated that K starvation-induced membrane dysfunction in maize roots primarily arose from impaired membrane permeability rather than from oxidative destruction of membrane lipids. K is known to regulate aquaporin activity and membrane protein stability through its effects on protein conformation and electrostatic interactions [28]. ROS accumulation suggested that the superior K efficiency of Ktm was not attributable to a generally stronger antioxidant capacity, but rather to a more precise regulation of ROS and detoxification. The root proteomic results provided a mechanistic explanation for this differential membrane integrity. In Ksm, respiratory burst oxidase homolog B (RBOH B), a key enzyme for apoplastic ROS signaling, was significantly downregulated (Table 4). This downregulation likely disrupts the early ROS-based signal transduction that normally triggers downstream antioxidant defenses. Concomitantly, Ksm also downregulated L-ascorbate peroxidase and dehydroascorbate reductase (Table 4), crippling the ascorbate–glutathione pathway, while its CAT activity showed no significant increase (Figure 7C). Consequently, although both genotypes showed increased accumulation of specific ROS species under K starvation, the predominant species differed critically: Ktm accumulated significantly more superoxide anion (O_2_^−^) (Figure 6C), whereas Ksm accumulated significantly more hydrogen peroxide (H_2_O_2_) (Figure 6D). We note that “total ROS” was not directly measured; rather, we measured specific ROS species (O_2_^−^ and H_2_O_2_) independently. It should be noted that total ROS levels were not directly measured; instead, specific ROS species (O_2_^−^ and H_2_O_2_) were quantified independently. Given that H_2_O_2_ possesses greater membrane-permeating capacity and toxicity than O_2_^−^, we propose that the failure to specifically scavenge H_2_O_2_, rather than total ROS burden, underlies the membrane leakage in Ksm.

By contrast, Ktm deployed a multi-layered, precisely orchestrated antioxidant network. It upregulated several peroxidases and peptide-methionine (R)-S-oxide reductase (Table 4). In addition, it importantly showed a significant increase in CAT activity (Figure 7C). This constellation of responses might have contributed to more effective H_2_O_2_ detoxification in Ktm, consistent with the observed maintenance of membrane permeability and minimizing lipid peroxidation damage. Recent studies have increasingly recognized “signal-execution coupling” as a pivotal factor governing stress resilience [29].

### 3.4. DAPs (Differentially Abundant Protein) Involved in Cell Wall Remodeling and Secondary Metabolism

Root system architecture and cell wall integrity are critical determinants of stress tolerance. Although total root length and surface area decreased in both genotypes, Ktm maintained a significantly larger total root volume under K starvation (Figure 2). Ktm specifically increased abundance of dirigent proteins (key regulators of lignin and lignan biosynthesis in plants [30]), probable cinnamyl alcohol dehydrogenase (CAD), glucan endo-1,3-β-D-glucosidase, and GDSL esterase/lipases (Table 5). The upregulation of dirigent proteins and CAD in Ktm was consistent with enhanced lignin biosynthetic potential, which might have contributed to cell wall reinforcement. Together, these proteomic changes were consistent with enhanced cell wall reinforcement and apoplastic sealing in Ktm. This might have contributed to the maintenance of normal electrolyte conductance in Ktm under K deprived conditions (Figure 6B), although direct measurements of cell wall composition and lignin content would have been required to confirm this interpretation. Conversely, Ksm decreased beta-expansin 1a (Table 5), a key cell wall-loosening protein essential for root elongation [31,32]. However, since root length and surface area decreased in both genotypes (Figure 2A,C), the downregulation of beta-expansin alone could not mechanistically account for the compromised root morphology in Ksm. Additional factors, including other cell wall-modifying proteins and hormonal regulation, likely contributed to the observed root morphological responses. Ksm also downregulated cell wall synthesis enzymes (glycosyltransferase and sulfotransferase) while showing increased abundance of several defense-related proteins (lipoxygenase, isoflavone reductase, and cytochrome P450). These changes might have indicated activation of chemical defense pathways in Ksm, although direct measurements of defense metabolites would have been required for confirmation.

**Figure 10:**
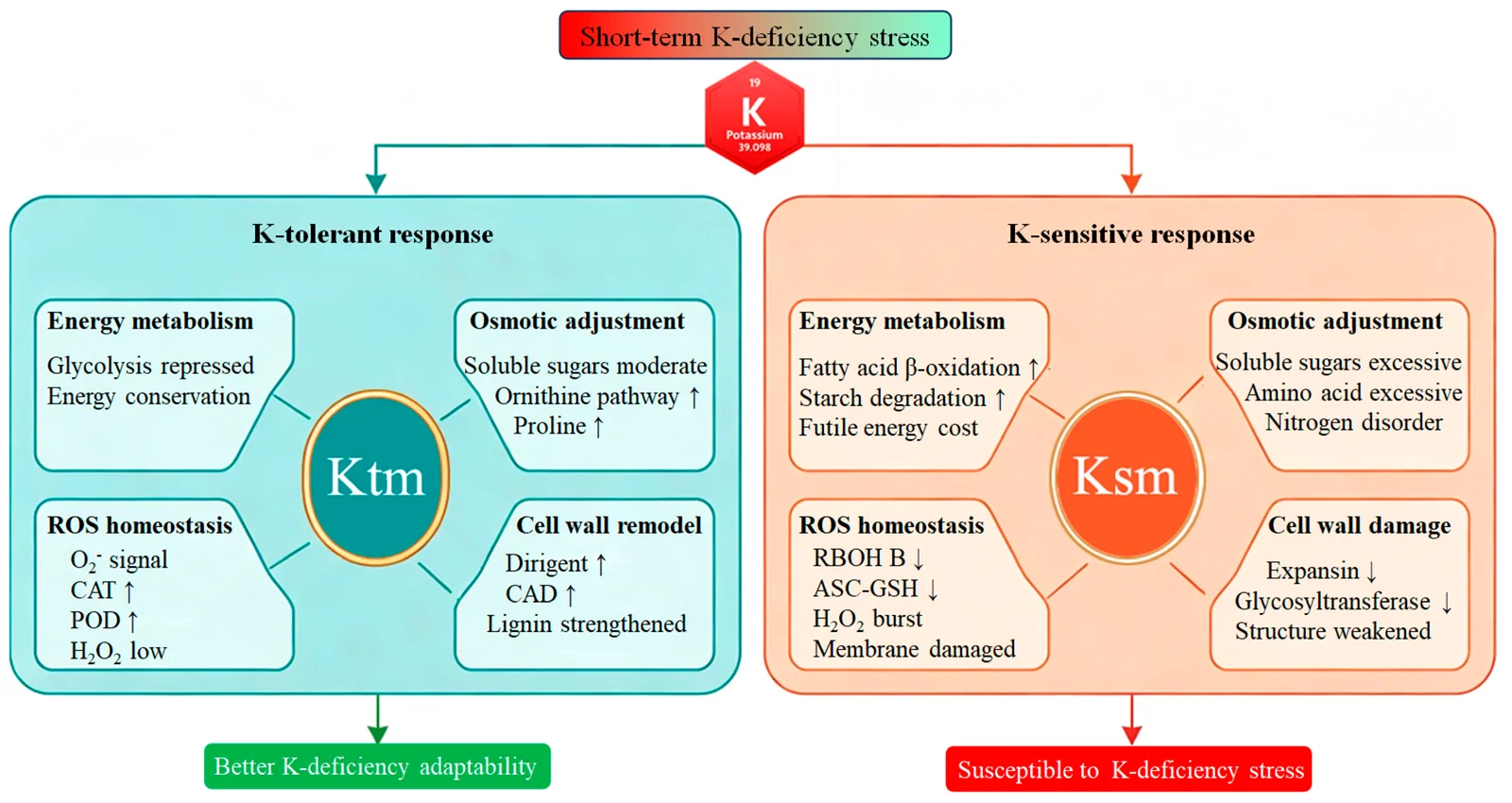
A conceptual model illustrating the mechanistic differences underlying short-term K-deficiency adaptation between K-tolerant (Ktm) and K-sensitive (Ksm) maize genotypes. CAD, cinnamyl alcohol dehydrogenase; CAT, catalase; POD, peroxidase; RBOH B, respiratory burst oxidase homolog B; ASC-GSH, ascorbate–glutathione cycle. Here, ↑ and ↓ represent upregulated and downregulated, respectively.

Based on the integrative physiological and proteomic evidence presented above, a conceptual model of genotype-specific short-term K-deficiency adaptation was proposed (Figure 10). Ktm showed coordinated changes consistent with energy conservation (suppressed glycolysis and costly processes such as ribosomal biogenesis and methyl cycling), redox homeostasis (enhanced CAT/POD activities, lowered H_2_O_2_, and maintained membrane permeability and root vitality), and cell wall reinforcement (upregulated dirigent and CAD). In contrast, Ksm exhibited upregulation of translational and methyl cycle machinery, impaired glutathione-dependent H_2_O_2_ detoxification, and compromised cell wall remodeling, coinciding with H_2_O_2_ accumulation, membrane dysfunction, and reduced root vitality. These patterns suggested that K tolerance was associated with coordinated metabolic repression, redox balance, and structural reinforcement rather than a single protective mechanism.

Several limitations of this study should be acknowledged. First, our findings are derived from a short-term (3-day) K-starvation experiment under controlled hydroponic conditions. Longer-term responses, field conditions, or different developmental stages may involve additional mechanisms not captured here. Second, our proteomic analysis was limited to root tissues; systemic responses involving shoot–root communication were not examined. Third, while we identified many DAPs, we did not measure corresponding metabolites or enzyme activities for all identified proteins, nor did we assess protein synthesis or degradation rates. Fourth, our conclusions regarding lignin biosynthesis, polyamine metabolism, and chemical defense are inferred from protein abundance changes and require direct biochemical validation. Finally, the absence of multiple testing correction in our proteomic analysis may have increased the risk of false positives; candidate DAPs identified here should be validated using targeted quantitative methods (e.g., PRM or Western blot) in future studies.

## 4. Materials and Methods

### 4.1. Plants Growth

The two maize inbred lines, Ktm (K tolerant) and Ksm (K sensitive), were obtained from the Maize Research Team of Shenyang Agricultural University. These lines were previously selected based on their contrasting phenotypic responses to low-K stress in preliminary field screenings, with Ktm showing superior root growth and biomass maintenance under K-limited conditions, while Ksm exhibited pronounced growth retardation and leaf chlorosis. The seeds of potassium-tolerant maize (Ktm) and potassium-sensitive maize (Ksm) were surface sterilized with 7% sodium hypochlorite solution for 10 min. Then, the seeds were thoroughly rinsed with deionized water to remove the residual sodium hypochlorite. The sterilized seeds were primarily germinated in silica sand for one week. After one week of germination in silica sand, the endosperm was carefully separated from the emerged seedlings using sterilized forceps, taking care not to damage the root–shoot junction or the primary root. Only seedlings with intact root systems and uniform size were selected for transplantation. The seedlings were carefully transplanted into an opaque plastic box (50 cm × 35 cm × 15 cm) containing 22 L nutrient solution (pH 6.0). Each opaque plastic box containing 22 L nutrient solution was considered one experimental unit, and randomization was performed. The complete nutrient solution contained 2000 μM Ca(NO_3_)_2_, 1250 μM KCl, 1000 μM MgSO_4_, 500 μM NH_4_H_2_PO_4_, 100 μM EDTA-Fe, 115 μM H_3_BO_3_, 22.5 μM MnCl_2_, 0.16 μM CuSO_4_, 0.75 μM ZnSO_4_, and 0.182 μM (NH_4_)_6_Mo_7_O_24_. To maintain the stability of K^+^ supply during plant growth, the K^+^ concentration in culture solution was monitored daily and replenished to 1.25 mM. The seedlings were propagated under long-day conditions (300–320 μmol m^−2^ s^−1^, 16 h light (24 °C)/8 h (22 °C) dark photoperiod and 60% relative humidity. For K starvation, 14-day-old plants were transferred to a K-free nutrient solution (0 mM KCl, −K) for another 3 days. The seedlings were still grown under complete nutrient solution (1.25 mM KCl, +K) as a control. Roots and shoots were respectively collected 3 days after K starvation. The sample was immediately chilled in liquid nitrogen and stored at −80 °C for further use. Three biological replicates were used for the comparative analysis of physiological traits. Each treatment was replicated four times for proteomic analyses, with each replicate consisting of 10 seedlings.

### 4.2. Biomass and Mineral Elements Assay

After washing with distilled water, the surface moisture of roots and shoots was blotted dry with absorbent paper. Subsequently, the roots and shoots after blotting dry were weighed to obtain the fresh weight (FW). The roots and shoots were wrapped in kraft envelopes, baked at 105 °C for 15 min, and dried at 70 °C to constant weight, followed by measurement of the dry weight (DW).

Root and shoot samples (approximately 0.1 g DW) were pulverized and digested with H_2_SO_4_-H_2_O_2_. K, Na, and Ca concentrations were assayed using flame photometry. The amounts of K accumulated in roots and shoots were computed based on the measured K concentration and the dry weight of each plant part.

P (phosphorus) was measured according to the vanadomolybdate method. Concisely, 1.0 mL of digestion solution was transferred into a 10 mL volumetric flask, and 20 μL of 2,6-dinitrophenol indicator was added. Gradually add 6 M NaOH until a faint yellow color just appeared. Then, 2 mL of 5 mM ammonium vanadomolybdate was added, and the volume was made up to 10 mL with distilled water. The solution was mixed thoroughly and allowed to stand for 15 min. The absorbance was then measured at 450 nm using a UV–Vis spectrophotometer. KH_2_PO_4_ standard solutions at concentrations of 0, 2, 4, 6, 8, and 10 mg·L^−1^ were measured under the same conditions as described above to construct a standard curve.

### 4.3. Root Morphology and Root Vitality Assay

The roots were scanned with an EPSON V800 scanner (Seiko Epson Corp., Suwa, Japan) to obtain morphological measurements, including total root length, average root diameter, root surface area, and root volume. These parameters were then analyzed using the WinRHIZO Program (Regent Instruments Inc., Quebec, QC, Canada).

For root vitality assays, root tips (approximately 0.5 g FW) were incubated in a solution consisting of 5 mL of 0.4% TTC (triphenyltetrazolium chloride) and 5 mL of 1/15 M phosphate buffer (pH 7.0) at 37 °C for 1 h in the dark. The reaction was immediately stopped by adding 2 mL of 1 M sulfuric acid. The root tips were then blotted dry with filter paper and ground with quartz sand and 4 mL of ethyl acetate, and the homogenate was filtered into a fresh tube. The residue was washed twice with ethyl acetate, and the washings were combined with the filtrate. Finally, the combined extract was adjusted to a final volume of 10 mL with ethyl acetate, and its absorbance was measured at 485 nm. Root vitality (μg·g^−1^·h^−1^) = tetrazolium reduction amount (μg)/[root fresh weight (g) × time (h)].

### 4.4. Lipid Peroxidation and Electrolyte Leakage Assay

The content of malonaldehyde (MDA) was measured to evaluate the lipid peroxide level as described in our previous study [33].

The electrolyte leakage assay was performed according the alteration in conductivity [34].

### 4.5. Osmotic Regulatory Substances Assay

The content of soluble protein in roots and shoots was measured using the Coomassie brilliant blue G-250 agent. Briefly, 0.3 g (FW) plant tissues were completely ground with phosphate buffer. The homogenate was transferred to a graduated tube, made up to 10 mL with phosphate buffer. After filtration, 0.1 mL of the filtrate was mixed with 5 mL Coomassie brilliant blue G-250, and the absorbance of the solution was then measured at 595 nm.

For total free amino acid content measurement, 0.1 g (FW) plant tissues were completely ground with 0.5 mL 10% acetic acid. The homogenate was transferred to a graduated tube, made up to 10 mL with distilled water, and then incubated in a boiling water bath for 10 min. After filtration, 1 mL of the filtrate was mixed with 0.5 mL of phosphate buffer and 0.5 mL of 2% ninhydrin, and the mixture was incubated in a boiling water bath for 15 min. The sample was cooled quickly with cold water and shaken frequently, and the absorbance of the solution was then measured at 570 nm.

The contents of free proline and soluble sugar in seedlings were measured according to a previous study [34].

### 4.6. Protein Extraction and iTRAQ Analysis

Seedling roots (ca 0.3 g, FW) were ground to a fine powder in a mortar with liquid nitrogen and then rinsed with 80% cold acetone. After centrifugation (12,000× *g* for 3min at 4 °C), the pellet was resuspended in 0.8 mL of extraction buffer (0.1 M Tris-HCl, pH 8.8, 10 mM EDTA, 0.4% β-mercaptoethanol, and 0.9 M sucrose) and mixed thoroughly. Subsequently, 0.8 mL of Tris-phenol was added, and the mixture was vortexed for 1 min and incubated at 4 °C for 30 min. After centrifugation (12,000× *g* for 5 min at 4 °C), the upper aqueous phase was transferred to a new tube, and five volumes of 0.1 M ammonium acetate in methanol were added to precipitate the proteins overnight at −20 °C. After centrifugation (12,000× *g* for 3 min at 4 °C), the pellet was washed twice with methanol, twice with 80% acetone, and once with 70% ethanol. The protein concentration was measured using the Bradford method [35].

A total of 100 μg protein was reduced with 10 mM DTT (dithiothreitol) at 56 °C for 2 h, followed by alkylation with 55 mM iodoacetamide in the dark at 24 °C for 45 min. Finally, the protein was subjected to tryptic digestion (enzyme-to-protein ratio 1:20, *w*/*w*) at 37 °C for 12 h. The vacuum dried peptides were labeled with iTRAQ reagents 8-plex kit according to the manufacturer’s instructions (AB SCIEX Inc., Framingham, MA, USA). The 8-plex iTRAQ reagents (113–121) were assigned as follows: 113: Ktm +K (replicates 2); 114: Ktm −K (replicates 2); 115: Ksm +K (replicates 2); 116: Ksm −K (replicates 2); 117: Ktm +K (replicates 2); 118: Ktm −K (replicates 2); 119: Ksm +K (replicates 2); and 121: Ksm −K (replicates 2). Protein identification and iTRAQ quantification were performed with ProteinPilot™ Software 5.0 (AB SCIEX) as described in our previous study [36]. Zea_mays.B73_RefGen_v4.pep.all.fa was used for the database searching, and the mass tolerance was set to 0.05 Da. The identified proteins with matched peptides ≥ 2, confidence > 95%, and an FDR (false discovery rate) ≤ 1% were used to perform protein quantification. A two-tailed t-test was employed, and a *p*-value was calculated for each protein. Subsequently, protein abundance (−K/+K) above 1.5-fold change (*p* < 0.05) was termed as DAP (differentially abundant protein). DAPs detected in at least three replicates of four biological replicates were used for further analysis.

STRING (version 11.0) (https://string-db.org/) was used for protein–protein interaction analysis with the *Zea mays* database, applying a medium confidence interaction score threshold of 0.4, with GO (Gene Ontology) enrichment and KEGG (Kyoto Encyclopedia of Genes and Genomes) pathway annotations [37].

### 4.7. Antioxidant System Assay

The content of superoxide anion (O_2_^−^) (nmol·g^−1^ FW) and hydrogen peroxide (H_2_O_2_) (nmol·g^−1^ FW) and the activities of antioxidases (superoxide dismutase (SOD) (U·min^−1^·mg^−1^ protein), peroxidase (POD) (U·min^−1^·mg^−1^ protein), and catalase (CAT) (U·min^−1^·mg^−1^ protein)) were measured using assay kits according to the corresponding instructions (Suzhou Comin Biotechnology Co., Ltd., Suzhou, China, catalog numbers: SA-1-Y, H_2_O_2_-1-Y, SOD-1-Y, POD-2-Y, and CAT-1-Y, respectively).

### 4.8. Statistical Analysis

Experimental data are presented as the means and standard deviations (SDs). Each physiological parameter was examined with four biological replicates. All physiological data were analyzed using SAS 9.2 (SAS Institute, Cary, NC, USA) with the SAS PROC ANOVA LSD model to perform the analysis of variance. A value of *p* < 0.05 was considered to be statistically significant.

## 5. Conclusions

A comprehensive investigation combining physiological measurements with comparative proteomic analysis using two maize inbred lines with contrasting K-deficiency tolerance were conducted in the present study. The K-tolerant maize genotype (Ktm) adopted an energy conservation strategy via coordinated downregulation of glycolysis and energy-consuming processes such as ribosome biogenesis and methyl cycling. Meanwhile, Ktm activated ROS detoxification and lignin-related cell wall reinforcement by upregulating peroxidases, CAT, dirigent proteins, and CAD, which preserved redox balance and root structural integrity under K deficiency. Overall, maize tolerance to K deficiency depended on the coordinated reprogramming of energy metabolism, redox homeostasis, and cell wall reinforcement rather than a single protective mechanism. Further field validation and functional characterization of key proteins will deepen our understanding of K-deficiency adaptation and support sustainable maize production.

## Figures and Tables

**Table 1:** KEGG pathway enrichment analysis of differentially abundant proteins (DAPs) in Ktm and Ksm under K starvation.

No	Pathway ID	Description	Number of DAPs			
			Ktm ↑	Ktm ↓	Ksm ↑	Ksm ↓
1	zma01100	Metabolic pathways	15	19	22	19
2	zma01110	Biosynthesis of secondary metabolites	10	16	13	9
3	zma01200	Carbon metabolism	4	6	——	4
4	zma00280	Valine, leucine, and isoleucine degradation	2	——	2	——
5	zma00592	alpha-Linolenic acid metabolism	2	——	2	——
6	zma00940	Phenylpropanoid biosynthesis	3	——	——	——
7	zma00020	Citrate cycle (TCA cycle)	2	——	——	——
8	zma00010	Glycolysis/gluconeogenesis	——	10	——	4
9	zma01230	Biosynthesis of amino acids	——	6	5	——
10	zma03010	Ribosome	——	4	6	——
11	zma00270	Cysteine and methionine metabolism	——	3	5	——
12	zma00030	Pentose phosphate pathway	——	2	——	2
13	zma00051	Fructose and mannose metabolism	——	5	——	——
14	zma00710	Carbon fixation in photosynthetic organisms	——	3	——	——
15	zma00500	Starch and sucrose metabolism	——	3	——	——
16	zma00620	Pyruvate metabolism	——	2	——	——
17	zma00250	Alanine, aspartate, and glutamate metabolism	——	——	4	——
18	zma00450	Selenocompound metabolism	——	——	3	——
19	zma00910	Nitrogen metabolism	——	——	3	——
20	zma00071	Fatty acid degradation	——	——	3	——
21	zma00970	Aminoacyl-tRNA biosynthesis	——	——	3	——
22	zma01212	Fatty acid metabolism	——	——	3	——
23	zma00630	Glyoxylate and dicarboxylate metabolism	——	——	3	——
24	zma04146	Peroxisome	——	——	3	——
25	zma00591	Linoleic acid metabolism	——	——	2	——
26	zma01040	Biosynthesis of unsaturated fatty acids	——	——	2	——
27	zma00220	Arginine biosynthesis	——	——	2	——
28	zma03030	DNA replication	——	——	2	——
29	zma00520	Amino sugar and nucleotide sugar metabolism	——	——	——	4
30	zma00480	Glutathione metabolism	——	——	——	3
31	zma00350	Tyrosine metabolism	——	——	——	2
32	zma00053	Ascorbate and aldarate metabolism	——	——	——	2

Note: The up and down arrows after each genotype represent increased and decreased protein abundance under K deficiency, respectively. The dash line indicates no data.

**Table 2:** Differentially abundant proteins (DAPs) involved in energy metabolism.

No.	Accession No.	Protein Name	Fold Change	
			Ktm	Ksm
1	Q43264	Alcohol dehydrogenase 1	−13.06 ± 1.49	−3.91 ± 0.60
2	C0HHC4	Nucleoside diphosphate kinase	−1.90 ± 0.31	−1.96 ± 0.12
3	B6SMQ5	Triose phosphate isomerase	−6.65 ± 0.22	——
4	A0A3L6EJY2	Glyceraldehyde-3-phosphate dehydrogenase	−6.45 ± 0.51	——
5	B4FWP0	Fructose-bisphosphate aldolase	−5.63 ± 0.73	——
6	P26301	Enolase 1	−3.27 ± 0.79	——
7	B4F9G8	Pyruvate kinase	−3.16 ± 0.76	——
8	B7ZYR6	Pyrophosphate-fructose 6-phosphate 1-phosphotransferase subunit alpha	−2.93 ± 0.31	——
9	Q6XZ78	Fructokinase-2	−2.05 ± 0.39	——
10	Q8S4W9	Pyruvate decarboxylase	−5.53 ± 0.73	——
11	B6T856	L-lactate dehydrogenase	−2.62 ± 0.73	——
12	B4G0N0	Glucose-6-phosphate isomerase	——	−1.73 ± 0.12
13	B7ZYP6	Pyruvate, phosphate dikinase	——	−3.78 ± 0.65
14	B6UAK0	Probable 6-phosphogluconolactonase	——	−1.96 ± 0.38
15	K7VNE0	Phosphoenolpyruvate carboxykinase (ATP)	2.27 ± 0.27	1.83 ± 0.15
16	C0P848	Formate dehydrogenase 2, mitochondrial	6.00 ± 0.57	3.60 ± 0.18
17	B6SHX1	Malate dehydrogenase	2.41 ± 0.71	——
18	B4G066	Methylmalonate-semialdehyde dehydrogenase (CoA acylating)	2.17 ± 0.46	——
19	B4FFP2	Glutamate dehydrogenase	——	1.76 ± 0.09
20	K7WFV1	ATP synthase subunit gamma	——	2.69 ± 0.62
21	A0A096PQY2	Long-chain-fatty-acid-CoA ligase	——	1.72 ± 0.18
22	B6TIL6	Acetyl-CoA C-acyltransferase	——	1.66 ± 0.11
23	B5AMJ8	Alpha-1,4 glucan phosphorylase	——	3.06 ± 0.11

Note: The red and green represent increased and decreased in proteins abundance under K deficiency, respectively. The dash line represents the protein abundance with no significant changes in response to K deficiency.

**Table 3:** Differentially abundant proteins (DAPs) involved in osmotic adjustment.

No.	Accession No.	Protein Name	Fold Change	
			Ktm	Ksm
1	B4FFJ4	Alpha-galactosidase	1.96 ± 0.07	3.09 ± 0.86
2	B6TIJ2	Acetylornithine deacetylase	3.31 ± 0.55	——
3	A0A804PS61	Acetylornithine transaminase	3.18 ± 0.18	——
4	A0A804QHK0	Ferredoxin-dependent glutamate synthase, chloroplastic	——	1.79 ± 0.32
5	B6T0A9	Beta-fructofuranosidase 1	——	4.12 ± 0.19
6	B4F972	Glutamate decarboxylase	——	2.22 ± 0.37
7	Q7SIC9	Transketolase	——	2.74 ± 0.89
8	B5AMJ8	Alpha-1,4 glucan phosphorylase	——	3.06 ± 0.11
9	B6TE43	Glutamine synthetase	——	−3.42 ± 0.81

Note: The red and green represent increased and decreased protein abundance under K deficiency, respectively. The dash line represents the protein abundance with no significant changes in response to K deficiency.

**Table 4:** Differentially abundant proteins (DAPs) involved in redox regulation.

No.	Accession No.	Protein Name	Fold Change	
			Ktm	Ksm
1	B6T2C6	Peptide-methionine (R)-S-oxide reductase	3.47 ± 0.27	——
2	B6SRH9	Peroxidase	5.08 ± 0.20	——
3	B4FY83	Peroxidase	3.30 ± 0.37	——
4	B4FKV6	Peroxidase	——	−3.99 ± 0.18
5	Q5EUE0	Protein disulfide-isomerase	−3.34 ± 0.82	——
6	B4FH44	Thioredoxin	−1.73 ± 0.21	−1.87 ± 0.31
7	C0P5U8	Glutathione transferase	−2.69 ± 0.26	−2.63 ± 0.12
8	B4FTF8	Glutathione transferase	−2.41 ± 0.39	——
9	Q9FQC1	Glutathione transferase	——	−3.26 ± 0.33
10	B4FRX8	L-ascorbate peroxidase	——	−2.13 ± 0.52
11	B4FT31	Dehydroascorbate reductase	——	−3.42 ± 0.86
12	A0A804REF8	Respiratory burst oxidase homolog protein B	——	−4.49 ± 0.20

Note: The red and green represent increased and decreased protein abundance under K deficiency, respectively. The dash line represents the protein abundance with no significant changes in response to K deficiency.

**Table 5:** Differentially abundant proteins (DAPs) involved in cell wall remodeling and secondary mechanisms.

No.	Accession No.	Protein Name	Fold Change	
			Ktm	Ksm
1	B6UI69	Acyl-desaturase	−2.70 ± 0.45	−3.75 ± 0.85
2	B4G0K5	Hydroxyproline-rich glycoprotein family protein	−3.48 ± 0.44	——
3	B4F938	Oxygen-dependent coproporphyrinogen-III oxidase	−2.68 ± 0.32	——
4	B6UAB6	Beta-expansin 1a	——	−1.90 ± 0.43
5	B4FJA8	Glycosyltransferase	——	−2.92 ± 0.63
6	B6STJ6	Sulfotransferase	——	−3.17 ± 0.41
7	B6SUA1	O-methyltransferase ZRP4	——	−1.51 ± 0.01
8	B6THD2	Glucan endo-1,3-beta-D-glucosidase	3.01 ± 0.19	——
9	B6SYY0	Beta-fructofuranosidase, insoluble isoenzyme 7	3.46 ± 0.41	——
10	A0A1D6LHB1	Dirigent protein	4.24 ± 0.89	——
11	Q49HE1	12-oxo-phytodienoic acid reductase	2.42 ± 0.25	——
12	B6U7D8	Probable cinnamyl alcohol dehydrogenase	3.23 ± 0.52	——
13	B4FVN5	Probable cinnamyl alcohol dehydrogenase	——	2.93 ± 0.89
14	P93518	Chitinase	——	2.03 ± 0.28
15	A0A1D6E4T8	Fibronectin type III-like domain-containing protein	——	2.72 ± 0.78
16	Q8W0V2	linoleate 9S-lipoxygenase	——	5.23 ± 0.75
17	A0A1D6N521	Lipoxygenase	——	2.81 ± 0.62
18	B6TVC6	Isoflavone reductase IRL	——	3.86 ± 0.27
19	B6UC65	Cytochrome P450 CYP71C1	——	1.95 ± 0.27

Note: The red and green represent increased and decreased protein abundance under K deficiency, respectively. The dash line represents protein abundance with no significant changes in response to K deficiency.

## Data Availability

The original contributions presented in this study are included in the article. Further inquiries can be directed to the corresponding author Shuisen Chen.

## Supplementary Materials

The following supporting information can be downloaded at: https://www.mdpi.com/article/10.3390/ijms27188286/s1.

## Abbreviations

The following abbreviations are used in this manuscript:

CAD	Cinnamyl alcohol dehydrogenase
CAT	Catalase
DAPs	Differentially abundant proteins
DTT	Dithiothreitol
GO	Gene Ontology
K	Potassium
KEGG	Kyoto Encyclopedia of Genes and Genomes
Ksm	K-sensitive maize genotype
Ktm	K-tolerant maize genotype
iTRAQ	Isobaric tags for relative and absolute quantitation
POD	Peroxidase
ROS	Reactive oxygen species
SD	Standard deviation
SOD	Superoxide dismutase
